# Coaching to develop leadership for healthcare managers: a mixed-method systematic review protocol

**DOI:** 10.1186/s13643-022-01946-z

**Published:** 2022-04-13

**Authors:** Shuang Hu, Wenjun Chen, Huiping Hu, Wenqiu Huang, Jia Chen, Jiale Hu

**Affiliations:** 1 Xiangya School of Nursing, Central South University, Changsha, China; 2 School of Nursing, Changsha Medical University, Changsha, China; 3 School of Nursing, Faculty of Health Science, University of Ottawa, Ottawa, Ontario Canada; 4 Centre for Research on Health and Nursing, University of Ottawa, Ottawa, Ontario Canada; 5 Hunan Cancer Hospital, Changsha, China; 6 Department of Nurse Anesthesia, Virginia Commonwealth University, Richmond, 907 Floyd Ave, Richmond, 23284 USA

**Keywords:** Coaching, Healthcare manager, Leadership, Systematic review

## Abstract

**Background:**

An increasing number of interventions have focused on leadership development for healthcare managers, among which coaching is a common strategy. The purpose of the present systematic review is to synthesize evidence on the effect of coaching in developing leadership of healthcare managers.

**Methods and analysis:**

A literature search will be conducted in six English databases (MEDLINE (Ovid), CINAHL, Embase, Cochrane library, Nursing & Allied Health Premium, and Scopus) and four Chinese databases (Wanfang, CNKI, SinoMed, and VIP) from inception to April 1st, 2022. The titles, abstracts, and full texts of the studies will be screened by two independent researchers to determine their eligibility. The RoB 2, ROBINS-I, CASP, and MMAT will be applied to assess the quality of randomized trials, non-randomized studies, qualitative studies, and mixed-method studies, respectively. We will then extract the study characteristics, participant characteristics, and study outcomes of the reviewed papers. The Aims, Ingredients, Mechanism, and Delivery framework will be used to extract the components of coaching strategies. For quantitative data, a meta-analysis will be performed if sufficient data are available; otherwise, we will conduct a narrative synthesis. Thematic synthesis methods will be used for qualitative data analysis.

**Discussion:**

By conducting this systematic review, we expect to synthesize evidence regarding the components of coaching for leadership development among healthcare managers; the influence of coaching on leadership development among managers at the individual, unit-wide, or organizational level; and how managers view coaching as a leadership development strategy.

**Trial registration:**

PROSPERO registration number: CRD42020194290.

**Supplementary Information:**

The online version contains supplementary material available at 10.1186/s13643-022-01946-z.

## Background

Leadership is defined as “a process whereby an individual influences a group of individuals to achieve a common goal” (p. 5) [1]. The importance of leadership is well recognized in healthcare organizations [2, 3]. The uptake of healthcare leadership frameworks is growing in countries such as the UK, the USA, Canada, and Australia to guide the development of leadership in healthcare organizations and ensure that high-quality care is provided for all [4–7]. For example, the Scottish Social Services Leadership Strategy Leadership Capabilities Framework was adopted for clinical nursing leadership development in the UK [8], and another theoretical empowerment framework was used to design and deliver a leadership development program for front-line nurse leaders in Canada [9].

Leadership development in healthcare organizations occurs at almost all levels [6, 7]. Healthcare managers are the primary target group [10] because many researchers and healthcare professionals contend that leadership among these individuals plays a critical role in improving individual and organizational performance [11, 12], enhancing productivity [12, 13], and incorporating research evidence in practice [14–16]. Continuous improvements in these areas can thus have a decisive impact on the quality of medical services and patient outcomes [17–19]. For example, the results of a study conducted by Gifford et al. [20] showed that the enhancement of nurse managers’ leadership behaviors can benefit the facilitation of guideline implementation and therefore positively influence the care of patients. The leadership development of healthcare managers is essential for implementing and sustaining practice innovation, and ensuring high-quality patient- or client-related healthcare delivery within an increasingly complex healthcare system [6, 21–23]. Therefore, it is essential to adopt effective strategies when developing healthcare managers’ leadership to improve healthcare service delivery [6, 24].

Coaching was initially used in the private sector as a method of leadership development among managers [25]. In 2011, 93% of US-based global 100 companies used coaching as a leadership development method [26]. Coaching was first introduced in the healthcare field in the 1830s [27]. Since then, an increasing number of studies have shown that coaching has the potential to retain leadership talent, support succession planning, and assist healthcare professionals in meeting personal and organizational goals [19, 23, 28, 29]. Researchers have found that coaching is an important leadership development strategy and a valuable investment for the long-term success of healthcare organizations [30, 31].

The Center for Creative Leadership defined coaching as a mutually beneficial relationship [32] “in which the coachee collaborates with the coach to assess and understand the coachee and his or her leadership developmental tasks, challenge current constraints while exploring new possibilities, and to ensure accountability and support for sustaining development and reaching goals” [33]. The essential role of a coach is to provide a dynamic, goal-based learning context for coachees. In such a context, the way coachees view themselves and the effect of their behaviors on others continues to expand, enabling them to be more influential and powerful and consequently achieve their goals [34]. Coaching involves investment in self-awareness development and focuses on sustained behavior development, modification, and the broadening of coachees’ perspectives [35, 36].

Athanasopoulou and Dopson [37] presented an extensive systematic review of 117 studies that evaluated coaching interventions for organizational executives. Executive coaching, as defined by the authors, is a targeted and purposeful intervention that helps executives develop and maintain positive changes in their personal development and leadership behavior. The review showed that cognitive behavioral, solution-focused, and positive psychology/strength coaching approaches were the most frequently applied coaching methods. The majority of the included studies showed that coaching has a positive influence on coaches, coachees (i.e., executives), and sponsored organizations. However, flaws in the research design limited the generalizability of the study outcomes. For example, many of the case studies consisted of narratives about coaching interventions from one or more coaches without looking for patterns or variations across cases.

To date, even with the increasing focus on coaching in leadership development for healthcare managers, no studies have examined the components of coaching strategies or the effectiveness of coaching in developing leadership for healthcare managers [19, 24, 38–40]. This systematic review addresses this gap in the literature by synthesizing studies that report on the content and influence of coaching, specifically for healthcare managers, and explore evidence-based approaches to develop coaching for healthcare managers’ leadership development. The overall goal of the systematic review is to synthesize evidence on the effect of coaching in developing leadership of healthcare managers. The specific objectives are as following: (1) to identify the components (aims, ingredients, mechanism, and delivery methods) of coaching for leadership development among healthcare managers, (2) to determine the impact of coaching on leadership development outcomes, and (3) to describe the perceptions of participants of coaching for leadership development.

## Methods and analysis

### Study design

A systematic review will be conducted using the Cochrane systematic review method [41]. The protocol will follow the Preferred Reporting Items for Systematic Review and Meta-Analysis Protocols (PRISMA-P) guidelines [42].

### Eligible criteria

#### Population

The eligible population in this review includes healthcare managers, defined as individuals in managerial positions responsible for managing daily activities and long-term goals for a healthcare organization [43]. Specifically, healthcare managers are classified by their role at two hierarchical levels: middle and senior levels [44, 45]. Middle managers [44, 46] manage front-line clinical practice on a daily basis and play an important role in translating top-level policies, strategies, and resources into practical improvements. Turnover and shortages of personnel, engagement, motivation, and the results of the workplace are all closely associated with healthcare management. Senior managers [47] have executive positions that involve wider organizational or departmental operational activities for healthcare delivery and have titles such as chief executive, health facility/hospital administrator, director, senior executive, executive manager, or operating officer. Only studies in which more than 50% of the target population are healthcare managers will be included.

#### Interventions

Coaching is an intervention or component of a complex intervention for leadership development among healthcare managers, including but not limited to leadership knowledge, behaviors, skills, attitudes, or practices [35, 40, 48, 49].

#### Comparators

Interventions (coaching) will be compared with alternative interventions or usual practices. As this is a mixed-method review, intervention studies with no control group will also be included [29].

#### Outcomes

The included quantitative studies must include quantitative outcome measures of coaching at the manager (e.g., leadership knowledge, skills, and behaviors), organization (e.g., length of hospital stay), practitioner (e.g., staff’s job satisfaction), or patient level (e.g., patient satisfaction and health outcome). The included qualitative studies must specifically focus on managers’ leadership (i.e., knowledge, skills, and behaviors) that result from a coaching intervention or the perceptions of participants of coaching for leadership development.

#### Types of studies

We will include several types of studies: (1) quantitative studies—randomized and non-randomized controlled trials and observational studies, such as cohort, case control, and cross-sectional studies; (2) qualitative studies, including those based on direct observation, in-depth interviews, and focus group discussions, among others, and (3) mixed-method studies. Non-empirical studies, such as discussions, commentary papers, reviews, or meta-analyses, will be excluded.

### Search strategy

The search strategy will use a mix of controlled terminology (MeSH or Cumulative Index to Nursing and Allied Health Literature headings) and keywords to ensure adequate subject coverage based on the concepts of coaching, leadership, and healthcare managers. Limits will be applied to language (English and Chinese only). No limits will be applied to the date of publication. A literature search will be performed in the MEDLINE (Ovid), CINAHL, Embase, Cochrane Library, Scopus, Nursing & Allied Health Premium, China National Knowledge Infrastructure (CNKI), Wanfang, 维普中文科技期刊全文数据库 (VIP), and 中国生物医学文献服务系统 (SinoMed) databases from inception to April 1st, 2022. The search strategy will be developed in accordance with the Cochrane Handbook [41]. Chinese and English librarians will be consulted to identify additional databases and formulate the most appropriate search strategy. A proposed sample search strategy in MEDLINE (Ovid) is included as an additional file (see Additional file 2). After the MEDLINE strategy is finalized, it will be adapted to the other databases listed above. After completing the electronic database search, we will cross-check the reference lists of the included studies to identify additional studies. We will also search key websites, such as Google Scholar, the Canadian Physician Coaches Network, and the Health Coach Alliance, for grey literature [50, 51].

### Study screening and selection

There researchers (SH, WH, WC) will screen and select the studies. First, all studies will be imputed into Covidence, a screening and data extraction software working in partnership with Cochrane to improve the production and use of systematic reviews for health and well-being. Duplicates will be removed and the remaining studies will be screened in two stages. In the first stage, two researchers (SH and WH) will independently screen titles and abstracts; in the second stage, full-text screening will be conducted independently by two researchers (SH and WH). These two stages will be recorded on Covidence, including the reasons for exclusion. Any discrepancies in the selection process will first be discussed between the two researchers, and if no consensus is achieved, a third researcher (WC) will be consulted. The results of the search and the screening process will be presented in a study flow diagram in accordance with the PRISMA guidelines [52].

### Methodological quality

Two researchers (HJ and HH) will independently assess the methodological quality of the studies. Any disagreements will be resolved through discussions with WC.

### Quantitative studies

We will assess the methodological quality of randomized trials using the revised Cochrane Risk-of-Bias (RoB 2) tool [53] (see Additional file 3), which measures (1) methodological quality arising from the randomization process, (2) methodological quality due to deviations from the intended interventions, (3) missing outcome data, (4) methodological quality in measurement of the outcome, and (5) methodological quality in the selection of the reported result. For non-randomized trials, the Risk of Bias in Non-randomized Studies-of Interventions (ROBINS-I) tool [54] (see Additional file 4) will be applied to assess the methodological quality based on the following domains: (1) confounding, (2) selection of participants into the study, (3) classification of interventions, (4) deviation from intended interventions, (5) missing data, (6) measurement of outcomes, and (7) selection of reported results. We will assess each domain of bias and the overall methodological quality as high, low, or unclear and then present a “methodological quality” table for all studies to increase transparency.

### Qualitative studies

We will use the Critical Appraisal Skills Programme (CASP) checklist [55] (see Additional file 5) for each qualitative study. The CASP checklist includes ten questions that are used to assess three broad issues: Are the results of the study valid? What are the results? Will the results help locally? Specifically, the ten questions relate to the research aim, methodology, recruitment, data collection, relationship between researcher and participants, work ethics, findings, and value of the research. Each question can be answered with “yes,” “no,” or “cannot tell.” We will then present the methodological quality of the studies in a tabular format. This checklist has been recommended by the Cochrane Collaboration for qualitative literature [56].

### Mixed-method studies

We will use the Mixed Methods Appraisal Tool (MMAT) to assess the methodological quality of mixed-method studies (see Additional file 6) [57]. We will assess three sets of items based on the study design: the qualitative set, quantitative set (either the RCT, non-randomized studies, or quantitative descriptive studies), and mixed methods set. Each item is rated on a categorical scale (yes, no, or cannot tell) [57].

### Data extraction

We will develop a form using Microsoft Excel sheets for data extraction. Two researchers (SH and JC) will perform data extraction independently. Discrepancies will be resolved by discussion with a third researcher (WC). We will search for secondary publications or make requests to the authors if implementation data are not included in the published articles or there is missing data. The following information will be extracted:

Study characteristics: authors, year of publication, journal, language, country, study purpose, research design, data collection method(s), and instruments or tools used.

Population characteristics: profession (e.g., nurse, physician), number of coaches and coachees, age range, gender, ethnicity, position, and level of seniority (e.g., junior, mid-level, or senior).

### Coaching

The Aims, Ingredients, Mechanism, Delivery (AIMD) framework [58] (see Additional file 7) will be applied to organize the extraction of coaching: (1) aims (what the intervention is intended to achieve and for whom); (2) ingredients (what comprises the intervention); (3) mechanisms (how the intervention is proposed to work); and (4) delivery (how the intervention is delivered).

### Outcomes

These include outcomes related to the effectiveness of coaching and managers’ perspectives and their experience of coaching.

### Data synthesis

To identify the components of coaching (objective one), as described above, all data about the components of interventions will be categorized based on the AIMD framework. We will then systematically code the data and inductively develop themes as they emerge in each category. Themes will be based on the primary author’s descriptions within each study whenever possible.

For quantitative data (objective two), we will decide whether to perform a meta-analysis based on data heterogeneity. There are three types of heterogeneity: clinical, methodological, and statistical. For all studies with statistical heterogeneity, we will use a random-effects model to combine the study data. For all studies with methodological and/or statistical heterogeneity (1) if the heterogeneity is too large to be resolved, we will report data narratively and descriptively instead of conducting a meta-analysis; (2) we will conduct a meta-regression analysis and mixed effects model; and (3) we will conduct subgroup analysis. Finally, we will perform a sensitivity analysis on the results to determine their reliability. We will report heterogeneity for pooled studies in meta-analysis using H statistics (CI = 95%, *P* < 0.05) and *I*^2^ statistics (the larger the *I*^2^ statistics, the greater the heterogeneity; low, medium, and high levels of heterogeneity are indicated by *I*^2^ statistics of 25%, 50%, and 75%, respectively, and *I*^2^ > 50% indicates that there is obvious heterogeneity).

We will analyze qualitative data regarding the influence of coaching on leadership development outcomes (i.e., at the managerial, organizational, practitioner, and patient level) (objective two) and participants’ perceptions of coaching for developing leadership (objective three) using thematic synthesis methods [59], which involves the following four-step process: (1) become familiar with data through an in-depth reading of included papers, (2) code the extracted qualitative data line-by-line, (3) categorize these codes into descriptive themes, and (4) develop these descriptive themes into analytical themes. The data synthesis will be completed by both SH and WC to ensure a high level of interrater reliability for qualitative rigor. The results will be compared and discussed at the meetings between both authors. A third author will also independently compare both analyses, provide feedback, and solve any outstanding disputes.

### Confidence in cumulative evidence

The quality of supporting evidence will be assessed using the Grades of Recommendation, Assessment, Development and Evaluation (GRADE) framework [56]. The GRADE-CERQual (Confidence in the Evidence from Reviews of Qualitative Research) tool will be used to assess the confidence of the qualitative data synthesis findings. This will provide readers with a detailed assessment of the methodological strengths and weaknesses of each reviewed study, which can then be used to assess the validity of their key findings [60]. We will report the assessment results and highlight the findings with the highest quality in the final review manuscript.

### Amendments

This is an original research protocol, as opposed to an amendment to a previously completed protocol. If the protocol requires major amendments, the changes will be documented and updated via PROSPERO and stated in the final review manuscript.

### Patient and public involvement

No patient was involved.

## Discussion

This systematic review is expected to provide a detailed summary of the evidence on the components of coaching for leadership development among healthcare managers. It is also expected that the results of this study will reveal the influence of coaching on leadership development among managers at the individual, unit-wide, or organizational level, as well as how managers view coaching as a leadership development strategy. This could provide a reference for future leadership development interventions among healthcare managers. The interaction and contributions of team members, as outlined in this protocol, will ensure a robust and rigorous review process. The research findings will be published in a peer-reviewed journal and presented at conferences and scientific meetings to disseminate knowledge.

## Supplementary Information

**Additional file 1.** PRISMA-P checklist

**Additional file 2.** Sample search strategy.

**Additional file 3.** Revised RoB 2 tool.

**Additional file 4.** ROBINS-I assessment tool.

**Additional file 5.** CASP Qualitative Checklist.

**Additional file 6.** Mixed Methods Appraisal Tool (MMAT).

**Additional file 7.** The AIMD framework.

## Data Availability

Not applicable

## Abbreviations

AIMD: Aims, Ingredients, Mechanism, Delivery
CASP: Critical Appraisal Skills programme;
CERQual: Confidence in the Evidence from Reviews of Qualitative Research
CINAHL: Cumulative Index to Nursing and Allied Health Literature
CNKI: China National Knowledge Infrastructure
GRADE: Grades of Recommendation, Assessment, Development and Evaluation
MMAT: Mixed Method Appraisal Tool
PRISMA-P: Preferred reporting items for systematic review and meta-analysis protocols
RoB 2: Risk-of-Bias ROBINS-I Risk of Bias in Non-randomized Studies-of Interventions

## References

[1] Northouse PG (2013). Leadership theory and practice.

[2] Bolden R, Gosling J, Marturano A, Dennison P (2003). A review of leadership theory and competency frameworks. Centre for leadership studies, University of Exeter.

[3] Shannon E, Sebastian A (2018). Developing health leadership with Health LEADS Australia. Leadersh Health Serv.

[4] Dawson S, Garside P, Hudson R, Bicknell C (2009). The design and Establishment of the Leadership Council.

[5] Hewison A, Morrell K (2014). Leadership development in the English National Health Service: a counter narrative to inform policy. Int J Nurs Stud.

[6] West MAK, Loewenthal L, Eckert R, West T, Lee A (2015). Leadership and leadership development in health care:the evidence base.

[7] Lyons O, George R, Galante JR, Mafi A, Fordwoh T, Frich J, et al. Evidence-based medical leadership development: a systematic review. BMJ Leader. 2020;1-8. 10.1136/leader-2020-000360. 10.1136/leader-2020-000360 37850339

[8] Cable S, Graham E (2018). “Leading Better Care”: an evaluation of an accelerated coaching intervention for clinical nursing leadership development. J Nurs Manage.

[9] MacPhee M, Skelton-Green J, Bouthillette F, Suryaprakash N (2012). An empowerment framework for nursing leadership development: supporting evidence. J Adv Nurs.

[10] Whaley A, Gillis WE (2018). Leadership development programs for health care middle managers: An exploration of the top management team member perspective. Health Care Manage Rev.

[11] Walker AG, Smither JW (1999). A five-year study of upward feedback: What managers do with their results matters. Personnel Psychol.

[12] Cleary S, Toit AD, Scott V, Gilson L (2018). Enabling relational leadership in primary healthcare settings: lessons from the DIALHS collaboration. Health Policy Plan..

[13] James AH, Stacey-Emile G (2019). Action learning: staff development, implementing change, interdisciplinary working and leadership. Nurs Manag (Harrow)..

[14] Gifford WA, Squires JE, Angus DE, Ashley LA, Brosseau L, Craik JM (2018). Managerial leadership for research use in nursing and allied health care professions: a systematic review. Implement Sci..

[15] Hu J, Gifford W (2018). Leadership behaviours play a significant role in implementing evidence-based practice. J Clin Nurs..

[16] Gifford WA, Davies B, Edwards N, Graham ID (2006). Leadership strategies to influence the use of clinical practice guidelines. Nurs Leadersh (Tor Ont).

[17] Richardson A, Storr J (2010). Patient safety: a literature [corrected] review on the impact of nursing empowerment, leadership and collaboration. Int Nurs Rev..

[18] Spence Laschinger HKPRN, Leiter MPP (2006). Patient safety: a literative review on the impact of nursing empowerment, leadership and collaboration. J Nurs Admin.

[19] Le Comte L, McClelland B (2017). An evaluation of a leadership development coaching and mentoring programme. Leadersh Health Serv (Bradf Engl)..

[20] Gifford W, Davies B, Tourangeau A, Lefebre N (2011). Developing team leadership to facilitate guideline utilization: planning and evaluating a 3-month intervention strategy. J Nurs Manag..

[21] Edmonstone J (2014). Action learning for change: a practical guide for managers. Action Learn.

[22] Chen W, Chen J, Hu J, Zhao J, Zhang J, He G (2021). The professional activities of nurse managers in chinese hospitals: a cross-sectional survey in Hunan Province. J Nurs Manage.

[23] Phillips N, Byrne G (2013). Enhancing frontline clinical leadership in an acute hospital trust. J Clin Nurs..

[24] De Brun A, O'Donovan R, McAuliffe E (2019). Interventions to develop collectivistic leadership in healthcare settings: a systematic review. BMC Health Serv Res..

[25] Jones RJ, Woods SA, Guillaume YRF (2016). The effectiveness of workplace coaching: A meta-analysis of learning and performance outcomes from coaching. J Occupational Organ Psychol.

[26] Augustijnen M-T, Schnitzer G, Van Esbroeck R (2011). A model of executive coaching: a qualitative study. Int Coaching Psychol Rev.

[27] Ewart KD (1923). The coaching class-lts aims and accomplishments. Psychol Clin.

[28] McNally K, Lukens R (2006). Leadership development: an external-internal coaching partnership. J Nurs Adm..

[29] Bradd P, Travaglia J, Hayen A (2018). Developing allied health leaders to enhance person-centred healthcare. J Health Organ Manag..

[30] Jeans ME (2006). Strengthening mentorship for leadership development. Can J Nurs Leadership.

[31] Bureau DA, Lawhead J (2018). Assessing student leadership development from mentoring, coaching, and advising. New Dir Stud Leadersh..

[32] Batara N, Woolgar T (2017). The mentorship imperative for health leadership. Healthc Manage Forum..

[33] Ting S, Hart EW (2004). Formal coaching..

[34] O’Flaherty C, Everson J (2005). Coaching in leadership development. Brain-Based Executive Education Johannesburg: Knowres.

[35] Hastings LJ, Kane C (2018). Distinguishing mentoring, coaching, and advising for leadership development. New directions for student leadership..

[36] Korotov K. Coaching for leadership development. The SAGE handbook of coaching. 2016;11(3):139–58.

[37] Athanasopoulou A, Dopson S (2018). A systematic review of executive coaching outcomes: Is it the journey or the destination that matters the most?. Leadership Q.

[38] Law H, Aquilina R (2013). Developing a healthcare leadership coaching model using action research and systems approaches - a case study: Implementing an executive coaching programme to support nurse managers in achieving organisational objectives in Malta. Int Coach Psychol Rev.

[39] Tom E, van D.S., Hilde E. (2016). Educating for ethical leadership through web-based coaching. Nurs Ethics..

[40] Fielden SL, Davidson MJ, Sutherland VJ (2009). Innovations in coaching and mentoring: implications for nurse leadership development. Health Serv Manage Res..

[41] Higgins JPT, Thomas J, Chandler J, Cumpston M, Li T, Page MJ, et al. Cochrane Handbook for Systematic Reviews of Interventions version 6.3 (updated February 2022). Cochrane. 2022. Available from: www.training.cochrane.org/handbook.

[42] Moher D, Shamseer L, Clarke M, Ghersi D, Liberati A, Petticrew M (2015). Preferred reporting items for systematic review and meta-analysis protocols (PRISMA-P) 2015 statement. Syst Rev..

[43] Belrhiti Z, Booth A, Marchal B, Verstraeten R (2016). To what extent do site-based training, mentoring, and operational research improve district health system management and leadership in low- and middle-income countries: a systematic review protocol. Syst Rev..

[44] Hartviksen TA, Aspfors J, Uhrenfeldt L (2017). Experiences of healthcare middle managers in developing capacity and capability to manage complexity: a systematic review protocol. JBI Database System Rev Implement Rep..

[45] Health S.o.S.f. High Quality Care for All. NHS Next Stage Review Final Report: Stationery Office; 2008. p. 66.

[46] Bradley EH, Taylor LA, Cuellar CJ (2015). Management matters: a leverage point for health systems strengthening in global health. Int J Health Policy Manage.

[47] White S, Milburn B, Jodoin TF, Downey DM (2004). Survey of Employers: Health Care Organizations’ Senior Nurse Managers.

[48] McNamara MS, Fealy GM, Casey M, O'Connor T, Patton D, Doyle L (2014). Mentoring, coaching and action learning: interventions in a national clinical leadership development programme. J Clin Nurs..

[49] Westcott L (2016). How coaching can play a key role in the development of nurse managers. J Clin Nurs..

[50] Alliance H.C. Health Coach Alliance [cited Available from: https://www.healthcoachalliance.ca/

[51] Canadian Physician Coaches Network [cited Available from: https://www.coach4md.org/

[52] Moher D, Liberati A, Tetzlaff J, Altman DG, Group P (2009). Preferred reporting items for systematic reviews and meta-analyses: the PRISMA statement. Ann Intern Med..

[53] Sterne JAC, Savovic J, Page MJ, Elbers RG, Blencowe NS, Boutron I (2019). RoB 2: a revised tool for assessing risk of bias in randomised trials. BMJ..

[54] Sterne JA, Hernan MA, Reeves BC, Savovic J, Berkman ND, Viswanathan M (2016). ROBINS-I: a tool for assessing risk of bias in non-randomised studies of interventions. BMJ..

[55] CASP Qualitative Checklist [cited Available from: https://casp-uk.net/wp-content/uploads/2018/01/CASP-Qualitative-Checklist-2018.pdf

[56] Guyatt GH, Oxman AD, Vist GE, Kunz R, Falck-Ytter Y, Alonso-Coello P (2008). GRADE: an emerging consensus on rating quality of evidence and strength of recommendations. Bmj..

[57] Hong QN, Gonzalez-Reyes A, Pluye P. Improving the usefulness of a tool for appraising the quality of qualitative, quantitative and mixed methods studies, the Mixed Methods Appraisal Tool (MMAT). J Eval Clin Pract. 2018;24(3):459-67. 10.1111/jep.12884 29464873

[58] Bragge P, Grimshaw JM, Lokker C, Colquhoun H, Group A.W.W (2017). AIMD - a validated, simplified framework of interventions to promote and integrate evidence into health practices, systems, and policies. BMC Med Res Methodol..

[59] Thomas J, Harden A, Oakley A, Oliver S, Sutcliffe K, Rees R (2004). Integrating qualitative research with trials in systematic reviews. BMJ..

[60] Geerts JM, Goodall AH, Agius S (2020). Evidence-based leadership development for physicians: a systematic literature review. Soc Sci Med.

