# Supplementary material for: Coaching to develop leadership for healthcare managers: a mixed-method systematic review protocol
Source: Syst Rev. 2022 Apr 13;11:67. doi: 10.1186/s13643-022-01946-z (PMC9008960; doi:10.1186/s13643-022-01946-z)
Supplement: Supplementary file 1 — Additional file 1. PRISMA-P checklist [file 13643_2022_1946_MOESM1_ESM.pdf]

PRISMA-P 2015 Checklist

This checklist has been adapted for use with systematic review protocol submissions to BioMed Central journals from Table 3 in Moher D et al: Preferred reporting items for systematic review and meta-analysis protocols (PRISMA-P) 2015 statement. *Systematic Reviews* 2015 **4**:1

An Editorial from the Editors-in-Chief of *Systematic Reviews* details why this checklist was adapted - Moher D, Stewart L & Shekelle P: Implementing PRISMA-P: recommendations for prospective authors. *Systematic Reviews* 2016 **5**:15

Section/topic	#	Checklist item	Information reported		Line number(s)
			Yes	No	
**ADMINISTRATIVE INFORMATION**					
**Title**					
Identification	1a	Identify the report as a protocol of a systematic review	☑	☐	Page 1, line 1
Update	1b	If the protocol is for an update of a previous systematic review, identify as such	☐	☐	
**Registration**	2	If registered, provide the name of the registry (e.g., PROSPERO) and registration number in the Abstract	☑	☐	Page 2, line 25
**Authors**					
Contact	3a	Provide name, institutional affiliation, and e-mail address of all protocol authors; provide physical mailing address of corresponding author	☑	☐	Page 1, line 4-26
Contributions	3b	Describe contributions of protocol authors and identify the guarantor of the review	☑	☐	Page 12, line 16-20
**Amendments**	4	If the protocol represents an amendment of a previously completed or published protocol, identify as such and list changes; otherwise, state plan for documenting important protocol amendments	☑	☐	Page 11, line 2-5
**Support**					
Sources	5a	Indicate sources of financial or other support for the review	☑	☐	Page 12, line 13-15
Sponsor	5b	Provide name for the review funder and/or sponsor	☑	☐	Page 12, line 13-15
Role of sponsor/funder	5c	Describe roles of funder(s), sponsor(s), and/or institution(s), if any, in developing the protocol	☑	☐	Page 12, line 13-15
**INTRODUCTION**					

BioMed Central The Open Access Publisher

Section/topic	#	Checklist item	Information reported		Line number(s)
			Yes	No	
**Rationale**	6	Describe the rationale for the review in the context of what is already known	☑	☐	Page 3 & 4
**Objectives**	7	Provide an explicit statement of the question(s) the review will address with reference to participants, interventions, comparators, and outcomes (PICO)	☑	☐	Page 5, line 2-8
**METHODS**					
**Eligibility criteria**	8	Specify the study characteristics (e.g., PICO, study design, setting, time frame) and report characteristics (e.g., years considered, language, publication status) to be used as criteria for eligibility for the review	☑	☐	Page 5, line 15-27; page 6, line 1-24
**Information sources**	9	Describe all intended information sources (e.g., electronic databases, contact with study authors, trial registers, or other grey literature sources) with planned dates of coverage	☑	☐	Page 6, line 25-27; page 7, line 1-13
**Search strategy**	10	Present draft of search strategy to be used for at least one electronic database, including planned limits, such that it could be repeated	☑	☐	Page 6, line 25-27; page 7, line 1-13
STUDY RECORDS					
Data management	11a	Describe the mechanism(s) that will be used to manage records and data throughout the review	☑	☐	Page 7, line 17-19
Selection process	11b	State the process that will be used for selecting studies (e.g., two independent reviewers) through each phase of the review (i.e., screening, eligibility, and inclusion in meta-analysis)	☑	☐	Page 7, line 14-26
Data collection process	11c	Describe planned method of extracting data from reports (e.g., piloting forms, done independently, in duplicate), any processes for obtaining and confirming data from investigators	☑	☐	Page 9, line 1-18
**Data items**	12	List and define all variables for which data will be sought (e.g., PICO items, funding sources), any pre-planned data assumptions and simplifications	☑	☐	Page 5, line 2-8
**Outcomes and prioritization**	13	List and define all outcomes for which data will be sought, including prioritization of main and additional outcomes, with rationale	☑	☐	Page 5, line 2-8
**Risk of bias in individual studies**	14	Describe anticipated methods for assessing risk of bias of individual studies, including whether this will be done at the outcome or study level, or both; state how this information will be used in data synthesis	☑	☐	Page 8, 1-27
DATA					

Section/topic	#	Checklist item	Information reported		Line number(s)
			Yes	No	
**Synthesis**	15a	Describe criteria under which study data will be quantitatively synthesized	☑	☐	Page 9, line 19-27; page 10, 1-20
	15b	If data are appropriate for quantitative synthesis, describe planned summary measures, methods of handling data, and methods of combining data from studies, including any planned exploration of consistency (e.g., I^2, Kendall's tau)	☑	☐	Page 9, line 25-27; page 10, 1-10
	15c	Describe any proposed additional analyses (e.g., sensitivity or subgroup analyses, meta-regression)	☑	☐	Page 10, line 11-20
	15d	If quantitative synthesis is not appropriate, describe the type of summary planned	☑	☐	Page 10, 1-10
**Meta-bias(es)**	16	Specify any planned assessment of meta-bias(es) (e.g., publication bias across studies, selective reporting within studies)	☐	☐	
**Confidence in cumulative evidence**	17	Describe how the strength of the body of evidence will be assessed (e.g., GRADE)	☑	☐	Page 10, 21-27
