# Supplementary material for: Coaching to develop leadership for healthcare managers: a mixed-method systematic review protocol
Source: Syst Rev. 2022 Apr 13;11:67. doi: 10.1186/s13643-022-01946-z (PMC9008960; doi:10.1186/s13643-022-01946-z)
Supplement: Supplementary file 2 — Additional file 2. Sample search strategy. [file 13643_2022_1946_MOESM2_ESM.pdf]

Additional File 2 Sample search strategy for MEDLINE (Ovid)

1 Personnel Management/

2 exp Health Facility Administration/

3 exp Institutional Management Teams/

4 Health Personnel/

5 Nurse Administrators/

6 ((health adj personnel) or supervisor* leader or leaders or administrator* or manager* or executive* or (senior* adj (staff? or nurse*)) or director* or chief* or officer*).ti,ab,kw,kf.

7 1 or 2 or 3 or 4 or 5 or 6

8 coach*.ti,ab,kw,kf.

9 Leadership/

10 leadership.ti,ab,kw,kf.

11 9 or 10

12 7 and 8 and 11

Notes:

term/ indicates subject heading

exp term/ indicates exploded subject heading

* indicates truncation

adj indicates adjacency operator
