# Supplementary material for: Coaching to develop leadership for healthcare managers: a mixed-method systematic review protocol
Source: Syst Rev. 2022 Apr 13;11:67. doi: 10.1186/s13643-022-01946-z (PMC9008960; doi:10.1186/s13643-022-01946-z)
Supplement: Supplementary file 7 — Additional file 7. The AIMD framework. [file 13643_2022_1946_MOESM7_ESM.pdf]

The AIMD framework

Component	Description	Definition and considerations
Aims	What do you want your intervention to achieve and for whom?	This component relates to the objective and outcome of the intervention. Based on your endpoint, what are you measuring in whom? It could include consideration of proximal and intermediate outcomes, and process outcomes related to implementation.
Ingredients	What comprises the intervention?	These are the observable, replicable, and irreducible aspects of the intervention. To increase the detail specified, other taxonomies could be used in conjunction with the AIMD framework. This might include intervention taxonomies or reporting guidance.
Mechanism	How do you propose the intervention will work?	This refers to the pathways or processes by which it is proposed that an intervention effects change or which change comes into effect. As with ingredients, other taxonomies could be used in conjunction with AIMD to add detail. The proposed mechanism could be based on either theory or empirical evidence, and be made specific to the setting. The use of mechanism may change depending on if the framework is used for reporting or designing: why was the ingredient selected (design) and what is the pathway in which it worked (reporting).
Delivery	How will you deliver the intervention?	This encompasses logistical and practical information pertaining to intervention delivery, including mode (e.g. video, brochure); level (e.g. individual, team, population); dose, frequency, intensity; who’s delivering; and size of target group.
